# Genetic Polymorphism of 27 Y-STR Loci in the Western Kazakh Tribes from Kazakhstan and Karakalpakstan, Uzbekistan

**DOI:** 10.3390/genes13101826

**Published:** 2022-10-09

**Authors:** Yeldar Ashirbekov, Zhaxylyk Sabitov, Baglan Aidarov, Arman Abaildayev, Zukhra Junissova, Alena Cherusheva, Viktoriya V. Saidamarova, Kamalidin Sharipov, Yerlan Ramankulov, Maxat Zhabagin

**Affiliations:** 1 M. Aitkhozhin Institute of Molecular Biology and Biochemistry, Almaty 050000, Kazakhstan; 2 National Center for Biotechnology, Astana 010000, Kazakhstan; 3 L.N. Gumilyov Eurasian National University, Astana 010000, Kazakhstan; 4 Research Institute for Jochi Ulus Studies, Astana 010000, Kazakhstan; 5 Karaganda Academy of the Ministry of Internal Affairs of the Republic of Kazakhstan Named after Barimbek Beisenov, Karaganda 100000, Kazakhstan; 6 School of Sciences and Humanities, Nazarbayev University, Astana 010000, Kazakhstan

**Keywords:** Y-STR, haplotype diversity, Kazakh, central Asia, population genetics

## Abstract

Data on the genetic polymorphism of 27 Y-STR in Kazakhs of the Junior Zhuz has been presented and analyzed in relation to forensic features. A total of 464 representatives of the Western Kazakh tribes of Kazakhstan (Western Kazakhs, *n* = 405) and Uzbekistan (Karakalpakstan Kazakhs, *n* = 59) were examined by the Yfiler Plus set. The data are available in the YHRD under accession numbers YA006010 and YA006009. Genetic analysis (AMOVA and MDS) did not show significant differences between the two groups (Kazakhstan and Karakalpakstan Kazakhs) in terms of Y-chromosome diversity. Both groups are characterized by haplogroup C2a1a2 as a founder effect, which dominated two of the three tribes: Alimuly (67%), Baiuly (74.6%), and Zhetiru (25.8%). At the same time, the phylogenetic network for each tribe found its own clusters within C2a1a2. Western Kazakhs and Karakalpakstan Kazakhs present high values of unique haplotypes (84.44% and 96.61%), discrimination capacity (90.37% and 98.30%), and haplotype diversity (0.9991 and 0.9994). A set of 27 Y-STR loci distinguishes closely related individuals within the Western Kazakh tribes quite well. It is suitable for forensic application, and is also optimal for population genetics studies.

## 1. Introduction

Analysis of polymorphism of short tandem repeats of the Y-chromosome (Y-STR) has become one of the main methods of forensic casework analysis. However, the evidentiary value of this analysis in court practice necessitates the constant improvement of population databases and the biostatistical applications that are integrated into them [1]. The largest database available online is the Y-chromosome haplotype reference database (YHRD), which includes more than a hundred national databases. In addition, it implements biostatistical tools such as the estimation of haplotype centers for the discrete Laplace calculation, AMOVA, and MDS visualization [2]. The genetic polymorphism of Y-STR loci is distributed globally in a heterogeneous way, reflecting, on the one hand, the patrilocality of many populations and, on the other hand, complex historical and demographic events as well as the results of the expansion of paternal lines in various geographical areas. This differential pattern is presented in the YHRD as metapopulations, the use of which is recommended in the interpretation of Y-STR results in forensic analysis [3]. The Eurasian-Altaic Metapopulation is one of the least balanced in the YHRD, with inhabitants from Central Asian countries remaining underrepresented (https://yhrd.org/, version R67). There are no data for Kyrgyzstan, Tajikistan, or Turkmenistan. Uzbekistan has data (*n* = 176) for a minimal set of 12 Y-STR loci [4]. For a diverse set of 27 Y-STR loci, only data from Kazakhstan (*n* = 723) has been reported [5,6]. However, Kazakhstan is not evenly represented in the YHRD—only two haplotypes for 27 Y-STR loci are reported from Western Kazakhstan [5], which is clearly insufficient to explain the genetic diversity of Western Kazakhstan’s Y-chromosome.

Western Kazakhstan accounts for 16% of the country’s total population (the population of Kazakhstan at the beginning of 2022 was 19,122,423 people, according to https://stat.gov.kz/). The indigenous population consists of Kazakhs, who account for 70% of the country’s overall population (~13.3 million people). Kazakhs make up 87 percent of Western Kazakhstan’s population (~2.5 million people), and are primarily represented by three prominent tribes: Alimuly, Baiuly, and Zhetiru. These tribes comprise the historical socio-territorial community known as the “Junior Zhuz.” The Kazakh population consists of more than 20 large tribes that have historically formed three socio-territorial communities: senior, middle, and Junior Zhuz. An individual’s belonging to a tribe or clan is determined by patrilineal inheritance. For many Turkic-speaking populations of Central Asia, it is associated with Y-chromosome inheritance and is expressed by a strong founder effect for the clans [7,8,9]. At the same time, ~10% of the genetic variability calculated for 15 Y-STR loci between Kazakh individuals is due to variability between the social-territorial communities (three zhuzes) of the Kazakhs [10].

Previous studies of the genetic polymorphism of the Y-chromosome of the Kazakh tribes of Western Kazakhstan were limited to 17 Y-STR loci [9,10]. A study of Y-SNP markers reveals a high frequency of the haplogroup C2a1a2-M48 for the Alimuly and Baiuly tribes (77% and 71%, respectively) [9]. Phylogenetic analysis within it singles out the Y15552 subbranch characteristic of these tribes, which coincides with the genealogy of these tribes in terms of the lifetime of the common ancestor and traces their origin to the Golden Horde Emir Alau (XV century). However, according to 17 Y-STR loci, it was not possible to clearly distinguish between closely related individuals in order to clearly separate individual genera from each other, having a common origin from Alau. Regarding this connection, it is important to apply rapidly mutating (RM) Y-chromosome loci to the study of these tribes, which significantly improve the separation of male relative differentiation [11].

Based on historical and ethnographic data, the Junior Zhuz has lived in all of Western Kazakhstan. It extends from the watershed Irgiz-Tobol-Turgay-Mugodzhary to the eastern tip of the Caspian Sea and the lower reaches of the Urals in the north, and from the lower and middle reaches of the Syr Darya to the Urals and Tobol in the south. It covers a large area, including the Mangyshlak Peninsula, the northern part of the Ustyurt Plateau, the eastern part of the Caspian Lowland and the Obshchy Syrt Upland, the Embenskoe and the western part of the Turgai Plateau, the southern tip of the Ural Mountains, Mugodzhary, the northern part of the Turan Lowland, and the northern coast of the Aral Sea [12].

The land of the Junior Zhuz is adjacent to Karakalpakstan, which is an autonomous republic in Uzbekistan. It encompasses the entire northwestern region of Uzbekistan. According to the statistical department of the Republic of Karakalpakstan (http://karakalpakstan.uz/), there were about 2 million people living there at the beginning of 2021. Karakalpaks made up 36.9% of the population; Uzbeks made up 40.3%; Kazakhs made up 15.5%; and others made up 7.3%. The modern Kazakhs of Karakalpakstan are descended from the Alimuly, Baiuly, and Tabyn clans of Zhetiru, who were living in the area that is now Karakalpakstan in the 1840s [13]. No studies have been conducted on the Y-chromosomes of Karakalpakstan Kazakhs.

This study aims to present and analyze the original data on Y-chromosome genetic polymorphism in Western Kazakhstan and Karakalpakstan. It uses the 27 STR loci that are currently relevant, which include both standard and rapidly mutating (RM) loci. In addition, it seeks to update the national databases of Kazakhstan and Uzbekistan in the YHRD with new data relevant to forensic and kinship casework. In the field of genetic genealogy, the goal of this research is to find genetic connections between tribes and nearby populations.

## 2. Materials and Methods

### 2.1. Sample Collection

In order to conduct research, 464 saliva samples were collected from healthy male Kazakh volunteers who had not been connected to each other in at least three generations and were aware of their tribal and clan affiliation. There were 405 samples from Kazakhstan and 59 samples from Karakalpakstan (Uzbekistan). Alimuly (*n* = 103), Baiuly (*n* = 272), and Zhetiru (*n* = 89) constitute the generalized tribal sample. Before providing saliva samples, all participants were informed about the study’s goals and objectives, after which they could sign informed consent forms and fill out ethnographic questionnaires. All necessary documents for the study were reviewed and approved by the Ethics Committee of the National Center for Biotechnology (No2 of 1 August 2019) and the Ethics Committee of the Asfendiyarov Kazakh National Medical University for M. Aitkhozhin Institute of Molecular Biology and Biochemistry (#6 of 29 October 2012). The procedures used in this study followed the ethical guidelines and the Declaration of Helsinki.

### 2.2. DNA Extraction and Y-STR Fragment Analysis

The Wizard (R) Genomic DNA Purification Kit (Promega, Madison, WI, USA) was used to extract DNA from saliva samples, and the manufacturer’s protocols were followed. The DNA concentration was determined using a Quantus Fluorometer (Promega, Madison, WI, USA) and a QuantiFluor(R) ONE dsDNA System kit (Promega, Madison, WI, USA). The NanoDrop One device (Thermo Fisher Scientific, Waltham, MA, USA) was used to determine DNA quality. Amplification of 27 STR loci of the Y chromosome was performed using the Yfiler^®^ Plus PCR Kit (Thermo Fisher Scientifc, Waltham, MA, USA) on a SimpliAmp Thermal Cycler (Thermo Fisher Scientifc, Waltham, MA, USA). The Yfiler^®^ Plus PCR Kit contains 17 standard STR loci (DYS19, DYS385 a/b, DYS390, DYS437, DYS439, DYS448, DYS456, DYS435, Y-GATA-H4) as well as 10 rapidly mutating loci (DYF387S1 a/b, DYS449, DYS460, DYS481, DYS518, DYS533, DYS570, DYS576, DYS627). Amplicon fragmentary analysis was performed on an 8-capillary Applied Biosystems 3500 genetic analyzer (Thermo Fisher Scientifc, Waltham, MA, USA). Further, the results were analyzed using the GeneMapper IDx v.1.5 software. (Thermo Fisher Scientifc, Waltham, MA, USA) according to the reference allelic ladder. The haplotype data were submitted to the YHRD (http://www.yhrd.org, accessed on 8 July 2022) with the accession numbers YA006010 and YA006009. In order to contribute to the haplotype data, the laboratories passed the Quality Control Test of the YHRD (YC000343).

### 2.3. Statistical Analyses

Haplotype frequency was calculated using Arlequin program ver. 3.5 [14]. The number of distinct haplotypes, frequency of unique haplotypes, discrimination capacity, haplotype match probability, and haplotype diversity were calculated by direct counting. The haplotype diversities were computed as HD = n*(1 − ∑p_i_^2^)/(*n* − 1), where *n* is the population size and pi is the frequency of *i-th* haplotypes. The sum of squared observed haplotype frequencies was used to determine the haplotype match probability (HMP). The ratio between the total distinct haplotypes and the number of haplotypes was used to calculate discrimination capacity (DC). The belonging of haplotypes to haplogroups was assessed using the Nevgen Y-DNA haplogroup predictor software (https://www.nevgen.org/). Forensic parameters such as random match probability (RM), power of discrimination (PD), gene diversity (GD), polymorphism information content (PIC), power of exclusion (PE), typical paternity index (TPI), and frequency for each locus were calculated using the STRAF program [15]. Phylogenetic networks based on Y-STR haplotypes of the Y-chromosome were built using the Network 5 software and Network Publisher (fluxus-engineering.com; Fluxus Technology) [16]. The allele sizes for locus DYS389II were determined by the subtraction of DYS389I. Loci DYS385 a/b and DYF387S1a/b were excluded from network analyses. Locus-specific weights were given according to the mutation rate for Y-STRs [17]. The “AMOVA and MDS” tools from the YHRD website (https://yhrd.org/, accessed on 8 July 2022) were used to compute pairwise genetic distances (*R_ST_*). Multidimensional scaling (MDS) was visualized using the XLSTAT software (https://www.xlstat.com/, accessed on 28 September 2022).

## 3. Results and Discussion

### 3.1. Haplotype/allele Frequencies and Forensic Parameters

Results for 27 Y-STR haplotype distributions are presented in Supplementary Table S1 for 405 Western Kazakhs and in Supplementary Table S2 for 59 Karakalpakstan Kazakhs. In the Western Kazakhs sample, 366 distinct haplotypes were found (Supplementary Table S3), and in the Karakalpakstan Kazakhs sample, 58 distinct haplotypes were found (Supplementary Table S4). Among Western Kazakhs, there were 24 haplotypes that were common to at least two individuals, and the most common haplotype was found in eight individuals. Among Karakalpakstan Kazakhs, only one haplotype was found to be shared by two individuals. Accordingly, for the Karakalpakstan Kazakhs discrimination capacity (98.3%), haplotype match probability (0.0175) and haplotype diversity (0.9994) were higher than for Western Kazakhs—DC (90.37%), HMP (0.0034), HD (0.9991). The results obtained were compared with the data of General Kazakhs [5] and Northern Kazakhs [6] in Table 1. Western Kazakhs and Karakalpakstan Kazakhs show a higher proportion of unique haplotypes, discrimination capacity, and haplotype diversity. However, Western Kazakhs are characterized by the lowest haplotype match probability.

Outcomes for 27 Y-STR allele frequencies and forensic parameters are presented in Supplementary Tables S5 and S6 for 405 Western Kazakhs and in Supplementary Tables S7 and S8 for 59 Karakalpakstan Kazakhs. Y-STR profiling revealed 168 alleles at single-copy loci among Western Kazakhs and 120 alleles at single-copy loci among Karakalpakstan Kazakhs. For Western Kazakhs, the frequency of single-copy loci alleles varies from 0.002 to 0.904. Of the 27 loci, DYS391 was found to have the fewest allelic variants (*n* = 3), while the largest (*n* = 11) was found for DYS449, DYS458, DYS481, DYS518, and DYS627. Gene diversity (GD) in the Western Kazakh samples varies from 0.181 for DYS392 to 0.735 for DYS627. Among the standard loci, the largest GD was found in DYS19 (0.706); among the rapidly mutating loci, the smallest GD was found in DYS460 (0.553). For Karakalpakstan Kazakhs, the frequency of the occurrence of single-copy loci alleles varied from 0.02 to 0.90. The largest number of allelic variants (*n* = 10) was found for the DYS481 locus, and the smallest (*n* = 3) for the DYS391, DYS393, and DYS460 loci. Gene diversity (GD) in the Karakalpakstan Kazakh samples varied from 0.19 for DYS392 and DYS456 to 0.82 for DYS481. On average, the gene diversity of eight rapidly mutating single-copy loci (0.65) is higher than that of the fifteen standard single-copy loci (0.44). Results for multi-copy loci DYS385a/b and DYF387S1a/b are presented in Supplementary Table S6 and S8. For Western Kazakhs, there are 35 allele combinations with 11 different DYS385a/b alleles and 39 allele combinations with 10 different DYF387S1a/b alleles. For Karakalpakstan Kazakhs, there are 10 allele combinations with 7 different DYS385a/b alleles and 17 allele combinations with 9 different DYF387S1a/b alleles. Gene diversity (GD) in both samples is higher at the DYF387S1a/b locus. Abnormal allele results are presented in Supplementary Table S9. Null alleles were found in loci DYS448 for both Western Kazakhs (*n* = 64) and Karakalpakstan Kazakhs (*n* = 2). Microvariant alleles were found only in Western Kazakhs (*n* = 3) at the DYS458 locus. A total of 59 copy number variations were identified in Western Kazakhs (*n* = 59) and Karakalpakstan Kazakhs (*n* = 7) at the DYS19 locus. A copy number variation was also found in the DYF387S1 locus for Karakalpakstan Kazakhs (*n* = 1). All of the variants were confirmed by repeating experiments.

### 3.2. Genetic Differentiation among Populations

**Figure 1:**
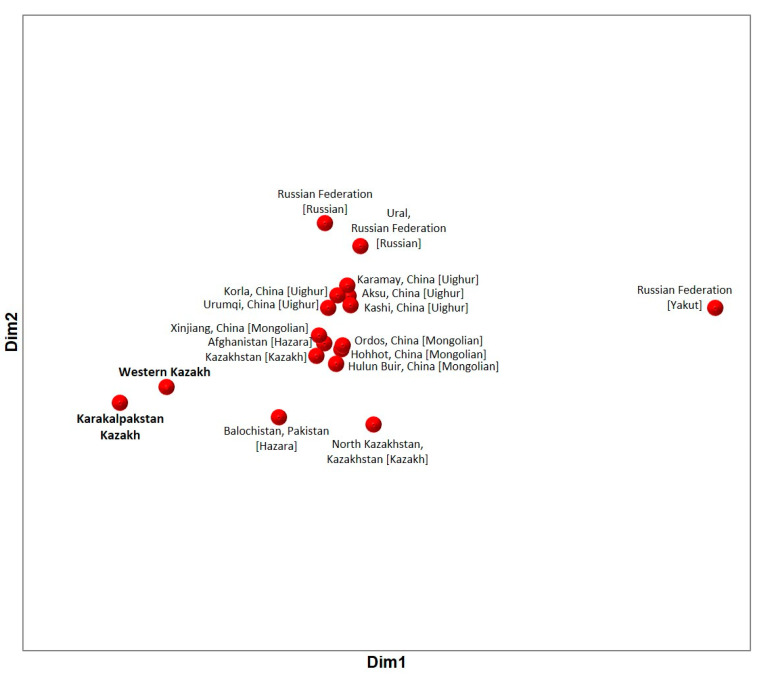
MDS plot based on *R_ST_* genetic distances between our samples and the neighboring populations based on 23 Y-STRs (stress = 0.074).

The pairwise genetic distance (*R_ST_*) results for 23 Y-STRs between Western Kazakhs and Karakalpakstan Kazakhs with neighboring populations are presented in Supplementary Table S10. As for neighboring populations, the available YHRD data was taken. It includes Kazakhstan [Kazakh] (341 haplotypes), North Kazakhstan, Kazakhstan [Kazakh] (382 haplotypes), Russian Federation [Russian] (895 haplotypes), Ural, Russian Federation [Russian] (91 haplotypes), Russian Federation [Yakut] (34 haplotypes), Hohhot, China [Mongolian] (240 haplotypes), Hulun Buir, China [Mongolian] (508 haplotypes), Ordos, China [Mongolian] (213 haplotypes), Xinjiang, China [Mongolian] (182 haplotypes), Afghanistan [Hazara] (260 haplotypes), Balochistan, Pakistan [Hazara] (153 haplotypes), Aksu, China [Uighur] (150 haplotypes), Karamay, China [Uighur] (129 haplotypes), Kashi, China [Uighur] (77 haplotypes), Korla, China [Uighur] (141 haplotypes), and Urumqi, China [Uighur] (49 haplotypes) [5,6,18,19,20,21,22,23]. The MDS plot of the compared populations is shown in Figure 1. Western Kazakhs and Karakalpakstan Kazakhs are located at a smaller genetic distance (*R_ST_* = 0.014) between each other in comparison with other populations. The closest in terms of genetic distances is the total sample of Kazakhstan [Kazakh] (*R_ST_* = 0.1042 for Western Kazakhs and *R_ST_* = 0.1381 for Karakalpakstan Kazakhs), in which there are only two haplotypes from Western Kazakhstan. At the same time, North Kazakhstan, Kazakhstan [Kazakh] are genetically located farther (*R_ST_* = 0.2113 for Western Kazakh and *R_ST_* = 0.2582 for Karakalpakstan Kazakh) than Afghanistan [Hazara] (*R_ST_* = 0.1434 for Western Kazakhs and *R_ST_* = 0.1882 for Karakalpakstan Kazakhs), Xinjiang, China [Mongolian] (*R_ST_* = 0.1103 for Western Kazakhs and *R_ST_* = 0.1418 for Karakalpakstan Kazakhs), or Urumqi, China [Uighur] (*R_ST_* = 0.1651 for Western Kazakhs and *R_ST_* = 0.2315 for Karakalpakstan Kazakhs). Such results require a more detailed study of intrapopulation differences among Kazakhs.

### 3.3. Phylogenetic Analysis of Kazakh Clans

Genetic polymorphism of 27 Y-STR haplotypes were also reanalyzed within the same sample (*n* = 464) but between the three Kazakh tribes: Alimuly (*n* = 103), Baiuly (*n* = 272), and Zhetiru (*n* = 89). Results for 27 Y-STR haplotype distributions are presented in Supplementary Tables S11–S13. The number of distinct haplotypes, frequency of unique haplotypes, discrimination capacity, haplotype match probability, and haplotype diversity for Alimuly, Baiuly, and Zhetiru are presented in Table 2. The highest heterogeneity is typical for Zhetiru. Supplementary Tables S1 and S2 presents the prediction of Y-chromosome haplogroups for each haplotype. Their frequency of occurrence is presented in Supplementary Table S14. Haplogroup C2a1a2–M48 (~prediction C2 M217 misc.) was found to be the most frequent for Alimuly (67%), Baiuly (74.6%), and Zhetiru (25.8%), with a high share of prediction. The obtained data do not contradict the previously discovered founder effect for these tribes within the C2-F5485 subhaplogroup of the C2-M48 haplogroup [9]. The same haplogroup is the core for both Western Kazakhs (64%) and Karakalpakstan Kazakhs (68%).

**Figure 2:**
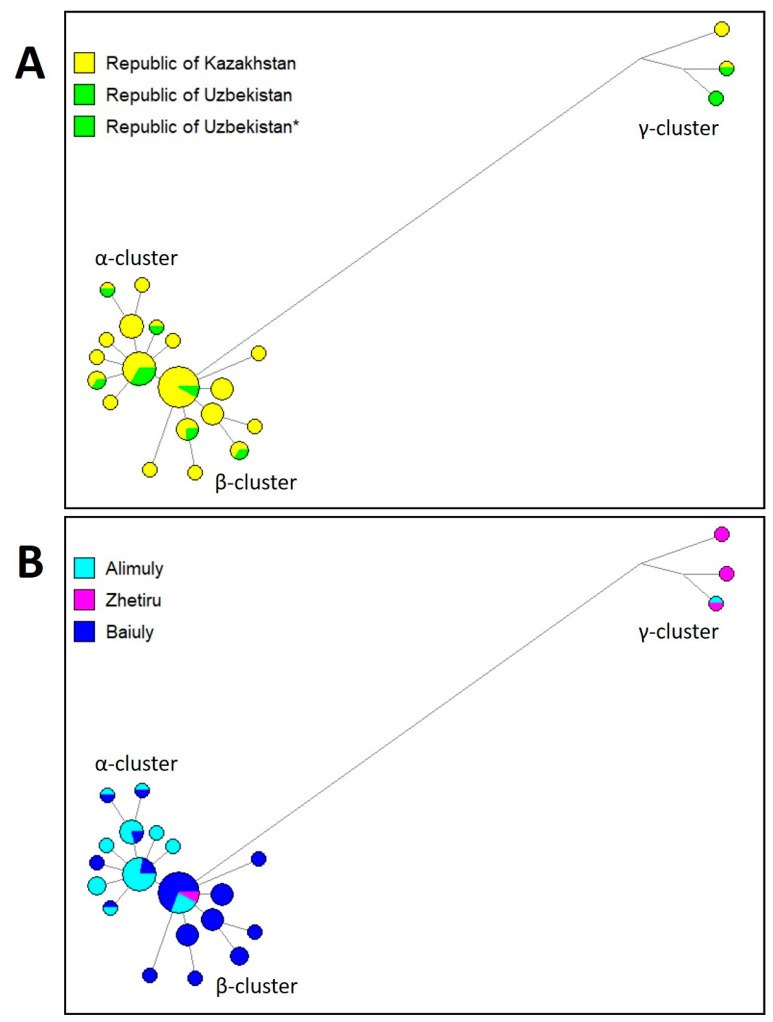
Phylogenetic Network of Western Kazakhs belonging to the C2a1a2–M48 haplogroup based in 23 Y-STRs. (**A**) Geographical affiliation. (**B**) Tribal affiliation.

The phylogenetic network of haplogroup C2a1a2 was based on 23 STR loci genotyped in 301 samples, except for haplotypes with a deletion at the DYS448 locus and with copy number variations at the DYS19 locus. Haplotypes with a deletion at the DYS448 locus are typical for the Baiuly tribe, in particular for the Berish clan (87%), while copy number variations in the DYS19 locus are more typical for Baiuly (80%). The phylogenetic network covers individuals from the Western Kazakhs group and the Karakalpakstan Kazakhs group. Geographical differentiation in Kazakhstan and Karakalpakstan (Uzbekistan) is not expressed (Figure 2A). Three clusters are distinguished in the phylogenetic network (Figure 2B), which largely coincide with the affiliation of individuals with the three tribes: Alimuly, Baiuly, and Zhetiru. In the phylogenetic network, α- and β-clusters are separated because the DYS570 locus has more of the allelic values 19, 18, and 17 in Baiuly (96%), and values 15, 16, and 17 in Alimuly (68%). The third γ-cluster diverges quite far from α- and β-clusters due to differences in allelic values in a number of loci (DYS19, DYS390, DYS391, DYS449, DYS460, DYS481, DYS533, DYS576, DYS627, DYS635, and DYS458). The third γ-cluster is mainly represented by the tribe Zhetiru, clan Tabyn. Thus, the results indicate significant differentiation across tribes.

## 4. Conclusions

In conclusion, this study presents data on the genetic polymorphism of the Y-chromosome of the western Kazakh tribes of Kazakhstan and Karakalpakstan, for 27 STR loci. The results indicate a common genetic origin of Western Kazakhs and Karakalpakstan Kazakhs, as well as their genetic distinction from Northern Kazakhs. The genetic links between the Alimuly, Baiuly, and Zhetiru tribes are expressed in their high differentiation along the paternal line. This indicates the relevance of research on the genetic polymorphism of the Y-chromosome in connection with the tribal structure of the Kazakh population. A wide set of 27 Y-STR loci distinguishes closely related male haplotypes quite well. New data added to YHRD (YA006010 and YA006009) will add diversity to the Eurasian-Altaic metapopulation. The new data will definitely help with forensic DNA applications and population genetic studies.

## Figures and Tables

**Table 1:** Comparison of forensic parameters among Kazakh populations based on 27 Y-STR haplotypes.

Population	Sample in Population	Number of Distinct Haplotypes	Frequency of Unique Haplotypes	Discrimi-Nation Capacity	Haplotype Match Probability	Haplotype Diversity
General Kazakh [5]	300	270	82%	0.9000	0.0042	0.9991
Northern Kazakh [6]	382	326	78%	0.8534	0.0044	0.9982
Western Kazakh	405	366	84.44%	0.9037	0.0034	0.9991
Karakalpakstan Kazakh	59	58	96.61%	0.9830	0.0175	0.9994

**Table 2:** Comparison of forensic parameters among Kazakh tribes based on 27 Y-STR haplotypes.

Tribe	Sample in Tribe	Number of Distinct Haplotypes	Frequency of Unique Haplotypes	Discrimi-Nation Capacity	Haplotype Match Probability	Haplotype Diversity
Alimuly	103	92	84.46%	0.8932	0.0137	0.9960
Baiuly	272	252	87.13%	0.9265	0.0044	0.9992
Zhetiru	89	85	91%	0.9550	0.0122	0.9990

## Data Availability

Not applicable.

## Supplementary Materials

The following supporting information can be downloaded at: https://www.mdpi.com/article/10.3390/genes13101826/s1. Table S1: The haplotype distributions of 27 Y-chromosomal STRs in the Western Kazakh tribes from Kazakhstan (*n* = 405). Table S2: The haplotype distributions of 27 Y-chromosomal STRs in the Western Kazakh tribes from Uzbekistan (*n* = 59). Table S3: The haplotype frequencies of 27 Y-chromosomal STRs in the Western Kazakh tribes from Kazakhstan (*n* = 405). Table S4: The haplotype frequencies of 27 Y-chromosomal STRs in the Western Kazakh tribes from Uzbekistan (*n* = 59). Table S5: Allele frequencies and forensic parameters values for 23 single-locus Y-STRs in the Western Kazakh tribes from Kazakhstan (*n* = 405). Table S6: Allelic combination frequencies and forensic parameters values for DYS385a/b and DYF387S1 in the Western Kazakh tribes from Kazakhstan (*n* = 405). Table S7: Allele frequencies and forensic parameters values for 23 single-locus Y-STRs in the Western Kazakh tribes from Uzbekistan (*n* = 59). Table S8: Allelic combination frequencies and forensic parameters values for DYS385a/b and DYF387S1 in the Western Kazakh tribes from Uzbekistan (*n* = 59). Table S9: Non-consensual alleles. Table S10: Pairwise genetic distance (*R_ST_*) between studied population and the Kazakh populations used for comparison from YHRD. Table S11: The haplotype frequencies of 27 Y-chromosomal STRs in the Alimuly tribe (*n* = 103). Table S12: The haplotype frequencies of 27 Y-chromosomal STRs in the Baiuly tribe (*n* = 272). Table S13: The haplotype frequencies of 27 Y-chromosomal STRs in the Zhetiru (*n* = 89). Table S14: The haplogroup frequencies in the Alimuly, Baiuly, and Zhetiru tribes by prediction.
